# Effects of applying blended learning based on the ADDIE model in nursing staff training on improving theoretical and practical operational aspects

**DOI:** 10.3389/fmed.2024.1413032

**Published:** 2024-06-28

**Authors:** Runfen Luo, Jing Li, Xiaoni Zhang, Dan Tian, Yi Zhang

**Affiliations:** ^1^Ear, Nose, Throat, Head and Neck Surgery Department of Xi'an Qinhuang Hospital, Xi’an, China; ^2^Anesthesia and Surgery Center of Xi'an Qinhuang Hospital, Xi’an, China; ^3^Interventional Diagnosis and Treatment Center of Xi'an Qinhuang Hospital, Xi’an, China

**Keywords:** nursing staff training, blended learning based on the ADDIE model, theory, practical operations, improvement, effect exploration

## Abstract

**Objective:**

To assess the impact of blended learning, based on the ADDIE model, on theoretical and practical aspects of nursing staff training.

**Methods:**

Retrospective analysis of data from 87 nursing staff members in Xi’an Qinhuang Hospital divided into control (*n* = 43) and observation (*n* = 44) groups. The control group received conventional training, while the observation group underwent blended learning. Comparative analysis included theoretical knowledge, practical skills, self-directed learning, critical thinking, and teaching satisfaction.

**Results:**

The observation group showed significantly higher theoretical knowledge, practical skills, self-directed learning, critical thinking, and teaching satisfaction compared to the control group (*p* < 0.05).

**Conclusion:**

Blended learning based on the ADDIE model enhances nursing staff training outcomes, improving theoretical knowledge, practical skills, self-directed learning, critical thinking, and teaching satisfaction. This approach presents a promising method for enhancing nursing education and warrants further implementation in clinical settings.

## 1 Introduction

Due to the ongoing issue of staffing shortages in the nursing field, coupled with an increase in patient demands, newly hired nursing staff often find themselves immediately immersed in demanding clinical work. However, the complexity and variability of clinical nursing require nurses to quickly acquire professional skills and critical thinking abilities (1). New nurses, referring to those who have recently graduated and entered clinical practice, require specialized training to help them adapt to their new roles. Research (2) indicates that new nurses need improvement in communication, critical thinking, leadership, and organizational skills, as deficiencies in these areas directly affect the quality of nursing care and patient safety. Therefore, effectively training new nurses to rapidly adapt to their new roles is an urgent issue to address. Despite the various training models currently available, the implementation of training often fails to fully consider the personal intentions and needs of new nurses (3, 4).

The ADDIE model is a training model designed and developed by the Educational Research Center at Florida State University, consisting of five phases: analysis, design, development, implementation, and evaluation (5). The model emphasizes systematic and targeted approaches, guided by the needs of learners, thereby avoiding the potential biases and blindness that may occur during training, reflecting an educational philosophy centered on the learner and ensuring training quality (6). Some studies on nursing training abroad (7, 8) have demonstrated the effectiveness and feasibility of this model. In the past, new nurse training mainly relied on traditional face-to-face and apprenticeship teaching methods. However, this approach often lacked diversity, systematicity, and homogeneity, resulting in insufficient mastery of professional knowledge and skills among new nurses. Blended learning is a teaching method that combines online and offline teaching (9). Building upon traditional face-to-face teaching, blended learning incorporates the advantages of the Internet and artificial intelligence technology to integrate “Internet +” deeply into theoretical and practical teaching, thereby breaking through the constraints of time and space in traditional teaching and fully leveraging the strengths of both online and offline teaching. Our research question is: What are the effects of blended learning based on the ADDIE model on improving theoretical knowledge, practical skills, self-directed learning abilities, critical thinking skills, and teaching satisfaction among nursing staff members?

Therefore, this study has designed a set of teaching plans based on the ADDIE model, employing a blended learning approach, aiming to explore its application effectiveness in standardized training for new nurses.

## 2 Objectives and methods

### 2.1 Study subjects

A retrospective analysis was conducted on the clinical data of 87 nursing staff members who participated in training between January 2022 and December 2023 in Xi’an Qinhuang Hospital. The patient selection was done by propensity score matching to mitigate the bias.

Inclusion criteria: (1) Nursing staff with less than 1 year of employment; (2) Nursing staff who have passed the professional qualification examination. Exclusion criteria: (1) Those unable to participate in training due to factors such as study abroad or conflicting vacations; (2) Those who did not pass the professional qualification examination; (3) Those unable to complete the training. Based on the nursing training mode received, they were divided into a control group (*n* = 43) and an observation group (*n* = 44). The control group received conventional nursing education training, while the observation group received blended learning training based on the ADDIE model.

**Figure 1:**
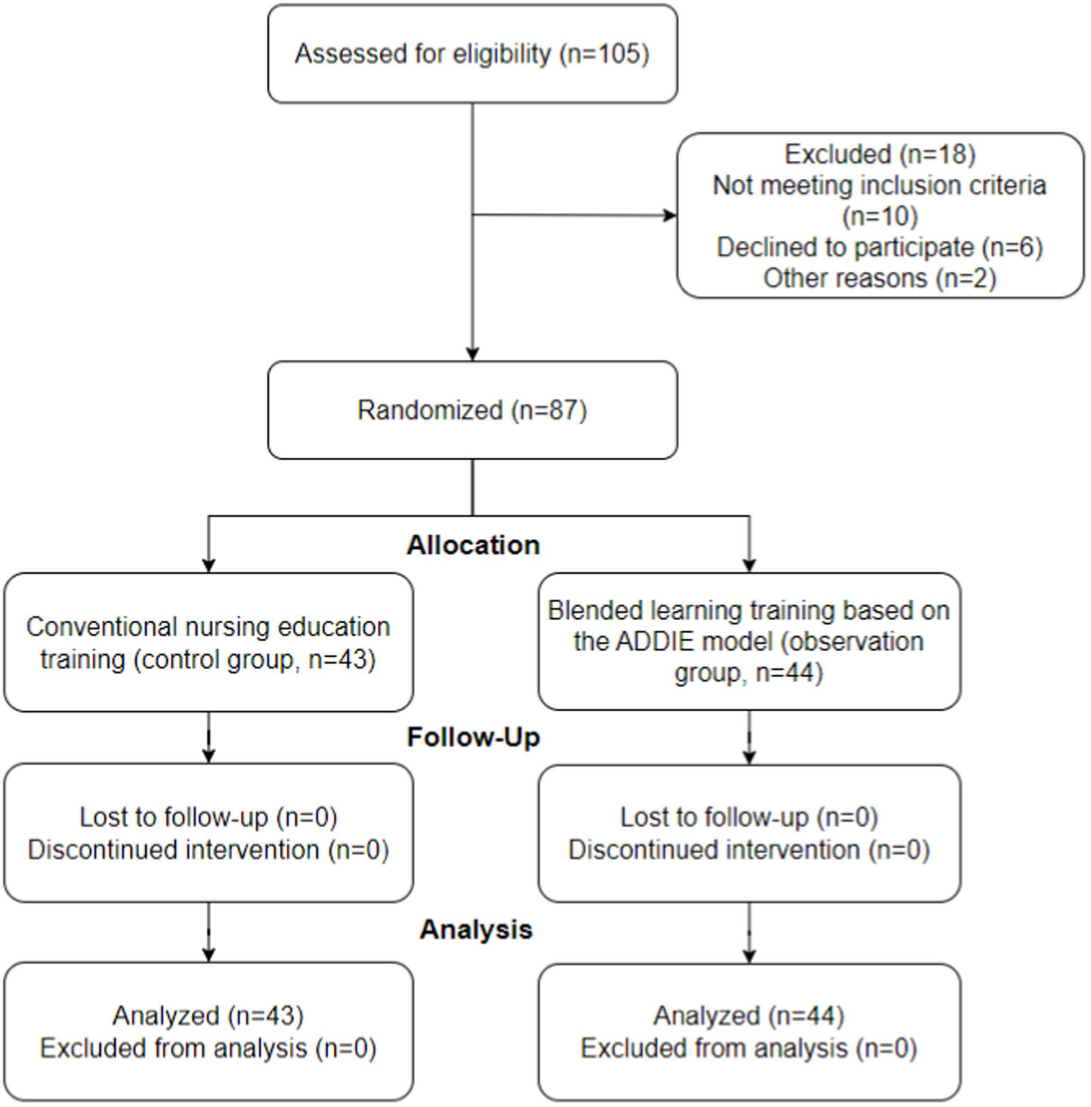
Flow chart of patient selection.

The flow chart of patient selection is presented in Figure 1.

### 2.2 Methods

#### 2.2.1 Control group

The control group received conventional nursing education training for a duration of 3 months. The training content was divided into two parts: (1) Initial training: This included an introduction to the hospital’s organizational structure, the responsibilities and work environment of various departments, basic nursing techniques, and the use of common medical equipment. (2) Practical training: New nurses were assigned to clinical teaching teachers for one-on-one field guidance. During the practical process, teachers demonstrated operations and explained steps, while new nurses mainly observed and learned. Teachers provided guidance during practice and answered questions as needed. Ultimately, new nurses needed to pass assessments to ensure they had mastered the relevant skills.

#### 2.2.2 Observation group

The observation group received blended learning based on the ADDIE model, which involved online and offline blended learning over a period of 3 months, conducted through the five steps of analysis, design, development, implementation, and evaluation. The specific content is as follows.

##### 2.2.2.1 Analysis

The analysis phase is a crucial part of the training plan and is essential for the success of the training work. A comprehensive analysis is conducted from three aspects: teaching objectives, teaching environment, and teaching content: (1) Teaching Objectives: The target of this training is newly hired nurses who lack clinical practice experience, have weak application abilities of knowledge, but are eager for their professional development. (2) Teaching Environment: The blended learning environment includes both online and offline aspects. The online environment utilizes modern network platforms, transforming the traditional teacher-centered model and providing students with more opportunities for self-directed learning. Traditional clinical teaching environments no longer meet the needs of modern nurses. Therefore, this study adopts scenario simulation teaching methods to stimulate students’ learning interests and enthusiasm through immersive experiences. (3) Teaching Content: Through literature review and survey analysis, it was found that the areas where new nurses most need improvement are nursing techniques, ability to observe and handle patient conditions, nursing documentation, and emergency procedures. Therefore, in designing the teaching content, we focus on cultivating new nurses’ practical skills, observation abilities, communication skills, emergency response capabilities, as well as problem analysis and solving abilities. The content covers common symptoms, common diseases and surgical procedures, symptom assessment, nursing techniques, emergency coordination, and emergency response procedures, aiming to integrate theory with practice to enhance the comprehensive qualities and coping abilities of new nurses.

##### 2.2.2.2 Design

In terms of teaching media, we plan to use online platforms as the main tool for knowledge transmission and adopt a blended learning model combining online and offline methods. We choose DingTalk as the main teaching platform to emphasize the “student-centered” teaching philosophy, aiming to enhance the guidance role of teachers in offline classrooms while promoting students’ self-directed learning abilities online. In terms of teaching methods, we will focus on the following aspects: (1) Introducing task-driven teaching methods: By assigning learning tasks before class, new nurses will learn in a problem-oriented manner, using inquiry-based tasks to stimulate and maintain their learning interests. (2) Using case analysis method: Allowing new nurses to actively think and understand the content learned through case analysis. (3) Utilizing mind maps for presentation of results: By integrating knowledge points from case analysis, promoting knowledge transfer and consolidation, and enhancing new nurses’ critical thinking abilities. (4) Applying scenario simulation methods: Conducting role-playing based on cases to maximize the reproduction of clinical situations, narrow the gap between theory and practice, and improve new nurses’ professional operating capabilities, emergency response abilities, and communication skills.

##### 2.2.2.3 Development

In the development phase, we focus on selecting suitable teaching resources and utilizing various auxiliary resources to systematically present the entire training framework and system. We select qualified senior nurses as clinical teaching mentors and form a new nurse training team together with the head nurses and department nurses. This team jointly develops and organizes the training outline to ensure consistency in training effectiveness. The team needs to complete the development and organization of online learning resources for new nurses before the training: (1) Developed teaching courseware divided by key points and difficulties, supplemented with images to enhance new nurses’ understanding of the knowledge points. (2) Produced short videos demonstrating common nursing techniques, with each video lasting less than 10 min, concise, vivid, and easily accessible for learning anytime and anywhere on mobile phones. For the display of specific details, new nurses can only have a preliminary understanding, and offline demonstrations by teachers are required. (3) Established a case library mainly based on common specialty cases, using relevant cases to introduce the theories and essential knowledge points involved in various specialties, organically integrating dry and abstract theories with practical applications. (4) Compiled a question bank for post-class assessments to help new nurses better grasp key and difficult issues, consolidate teaching effectiveness, and promote comprehensive learning among nurses.

##### 2.2.2.4 Implementation

In the context of “Internet +,” we have developed a comprehensive online and offline teaching optimization model integrating pre-class, in-class, and post-class stages: (1) In the pre-class preparation stage, clinical teachers use task-driven teaching to notify new nurses to review relevant knowledge points. They publish teaching courseware on the online platform for new nurses to learn independently. New nurses can complete their learning through various methods, such as consulting cases, asking clinical teachers for advice, searching for information online, and conducting clinical observations, while recording any questions they have. (2) During the in-class teaching stage, a combination of case analysis and scenario simulation teaching methods is adopted. Teachers select clinical cases for discussion based on the content that new nurses have independently learned. Through analogy with cases, nursing knowledge points are organically connected to establish a relatively complete knowledge system. Meanwhile, through scenario simulation teaching, cases are presented in simulated clinical scenarios to enhance new nurses’ learning enthusiasm, deepen their impressions, and cultivate practical skills. (3) In the post-class learning stage, new nurses engage in independent review, feedback on doubts from the class, and completion of homework. They can be advised and guided to use clinical cases as a starting point to organize mind maps, systematizing and organizing scattered knowledge to consolidate learning outcomes.

##### 2.2.2.5 Evaluation

Throughout the training process, the evaluation of learning and teaching effectiveness is always emphasized. Methods such as online questionnaires and offline assessments are employed to assess the improvement of new nurses’ abilities and their mastery of knowledge and skills. Additionally, through methods like questionnaire surveys or face-to-face interviews, the effectiveness of teaching implementation is understood, reflecting on various aspects of teaching design, and continuously improving the blended learning model of online and offline teaching through a virtuous cycle. This evaluation method not only helps to identify problems promptly but also promotes the continuous improvement and enhancement of the training model.

### 2.3 Observation indicators

#### 2.3.1 Levels of theoretical knowledge and practical skills

The nurse training group compiled assessment papers based on the training content, divided into theoretical and practical assessments, each with a maximum score of 100 points, and new nurses were required to complete the assessments face-to-face. (1) Theoretical Part: Questions were randomly selected from the question bank, including theoretical assessments and case analysis. A score of ≥80 points was considered passing. (2) Practical Part: Implementation of objective structured clinical examinations (OSCE) based on scenario cases. New nurses were randomly assigned one case for examination. A score of ≥90 points was considered passing. The examiners were composed of representatives from the head nurse, overall preceptor, and nursing unit leader, each conducting independent scoring.

#### 2.3.2 Self-directed learning abilities

The self-directed learning ability scale for nursing students was used to assess the self-directed learning abilities of new nurses. This scale consists of four dimensions: learning motivation, planning and implementation, self-management, and interpersonal communication, with a total of 20 items. Each item was scored on a Likert scale of 5 points, where “strongly agree” scored 5 points and “strongly disagree” scored 1 point. A higher total score indicates stronger self-directed learning abilities in nurses.

#### 2.3.3 Critical thinking abilities

The critical thinking ability measurement scale was used to evaluate the critical thinking abilities of new nurses. This scale consists of seven dimensions: seeking truth, open-mindedness, analytical skills, systematic skills, confidence in critical thinking, curiosity, and cognitive maturity.

#### 2.3.4 Teaching satisfaction

The hospital’s self-developed “Teaching Satisfaction Survey Questionnaire” was given to new nurses for evaluation. This questionnaire comprises 20 questions, and new nurses rate their satisfaction with nursing training and teaching content. Each question is scored out of 5 points, with a total score of <70 points indicating dissatisfaction, 70–89 points indicating satisfaction, and ≥90 points indicating very satisfied.

### 2.4 Statistical analysis

GraphPad Prism 8 (GraphPad Software, Inc., San Diego, California, United States) was used for graphing, and SPSS 22.0 (IBM, Chicago, Illinois, United States) was used for data analysis. For continuous data, the mean and standard deviation were used to describe the distribution, and statistical analysis was performed using t-tests or analysis of variance (ANOVA). For categorical data, frequencies and percentages were used to describe the distribution, and statistical analysis was performed using chi-square tests or Fisher’s exact tests. A *p*-value < 0.05 was considered statistically significant.

## 3 Results

### 3.1 Comparison of basic data

The basic data of the two groups of patients were comparable, with no significant differences in comparison (*p* > 0.05) (see Table 1 for details).

**Table 1:** Comparison of basic data.

	Control (*n* = 43)	Observation (*n* = 44)	*t*/*x*^2^	*p*
Gender	–	–	0.000	0.984
Male	2 (4.65%)	1 (2.27%)	–	–
Female	41 (95.35%)	43 (97.73%)	–	–
Mean age (years)	22.34 ± 0.95	21.93 ± 0.99	1.970	0.052
Educational level	–	–	0.283	0.594
Diploma	22 (51.16%)	20 (45.46%)	–	–
Bachelor’s degree	17 (39.54%)	19 (43.18%)	–	–
Master’s degree	4 (9.30%)	5 (11.36%)	–	–
Residence	–	–	0.109	0.740
Urban	19 (44.19%)	21 (47.73%)	–	–
Rural	24 (55.81%)	23 (52.27%)	–	–

### 3.2 Comparison of theoretical knowledge and practical skills

**Figure 2:**
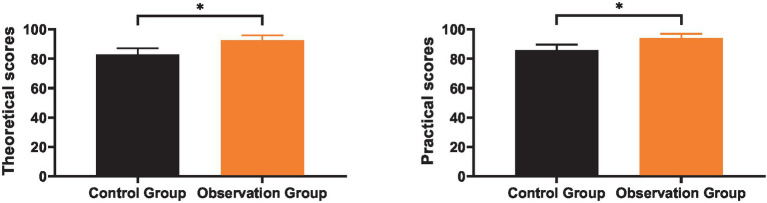
Comparison of theoretical knowledge and practical skills. * indicates a statistically significant difference with *p* < 0.05.

As shown in Figure 2, the mean theoretical knowledge level of the control group after training was (82.96 ± 4.13), and the practical skills level was (85.83 ± 3.76); the mean theoretical knowledge level of the observation group after training was (92.74 ± 3.18), and the practical skills level was (94.12 ± 2.93). After training, the theoretical knowledge level and practical skills level of the observation group were significantly higher than those of the control group (*p* < 0.05).

### 3.3 Comparison of self-learning abilities

**Figure 3:**
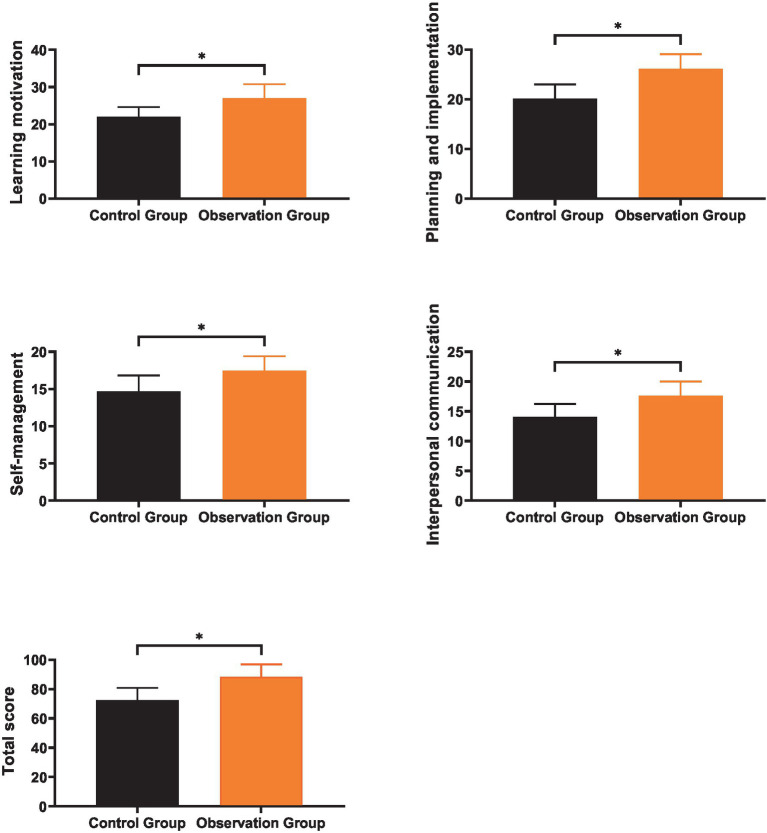
Comparison of self-learning abilities. * indicates a statistically significant difference with *p* < 0.05.

As shown in Figure 3, the mean levels of learning motivation, planning and implementation, self-management, and interpersonal communication of the control group after training were (21.97 ± 2.64), (20.13 ± 2.89), (14.67 ± 2.13), and (14.08 ± 2.16), respectively, with a total score of (72.39 ± 8.42); the mean levels of learning motivation, planning and implementation, self-management, and interpersonal communication of the observation group after training were (27.05 ± 3.72), (26.15 ± 2.94), (17.44 ± 1.95), and (17.65 ± 2.37), respectively, with a total score of (88.32 ± 8.56). After training, the scores of various dimensions (learning motivation, planning and implementation, self-management, interpersonal communication) and the total score of self-learning abilities of the observation group were significantly higher than those of the control group (*p* < 0.05).

### 3.4 Comparison of critical thinking abilities

**Figure 4:**
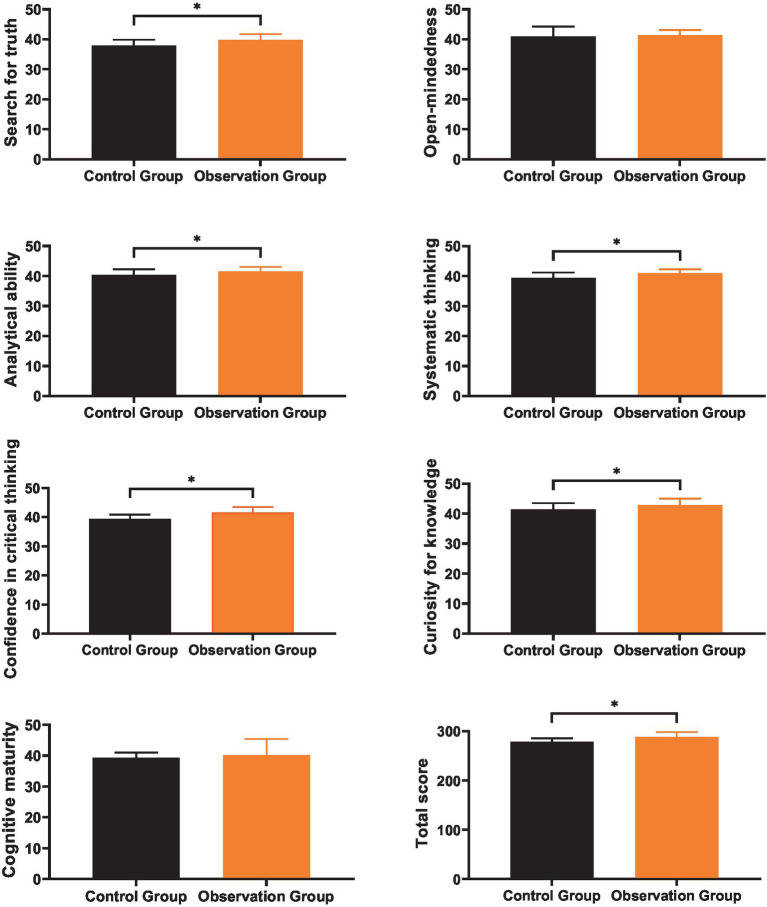
Comparison of critical thinking abilities. * indicates a statistically significant difference with *p* < 0.05.

As shown in Figure 4, the mean levels of seeking truth, open-mindedness, analytical ability, systematic thinking, confidence in critical thinking, curiosity for knowledge, and cognitive maturity of the control group after training were (37.85 ± 2.04), (40.96 ± 3.32), (40.35 ± 1.94), (39.45 ± 1.78), (39.37 ± 1.52), (41.39 ± 2.12), and (39.25 ± 1.78), respectively, with a total score of (278.56 ± 6.98); the mean levels of seeking truth, open-mindedness, analytical ability, systematic thinking, confidence in critical thinking, curiosity for knowledge, and cognitive maturity of the observation group after training were (39.79 ± 1.97), (41.31 ± 1.78), (41.48 ± 1.53), (40.99 ± 1.26), (41.56 ± 1.95), (42.83 ± 2.19), and (40.17 ± 5.22), respectively, with a total score of (288.11 ± 10.43). After training, the mean scores of various dimensions (seeking truth, analytical ability, systematic thinking, confidence in critical thinking, curiosity for knowledge) and the total score of critical thinking abilities of the observation group were significantly higher than those of the control group (*p* < 0.05); there was no significant difference between the two groups of nurses in open-mindedness and cognitive maturity scores (*p* > 0.05).

### 3.5 Comparison of teaching satisfaction

The teaching satisfaction of the control group was 81.40%, while that of the observation group was 97.73%. The teaching satisfaction of the observation group was significantly higher than that of the control group (*p* < 0.05) (see Table 2 for details).

**Table 2:** Comparison of teaching satisfaction.

Group	*n*	Dissatisfied	Satisfied	Very satisfied	Total satisfaction rate
Control	43	8 (18.60%)	19 (44.19%)	16 (37.21%)	35 (81.40%)
Observation	44	1 (2.27%)	16 (36.36%)	27 (61.36%)	43 (97.73%)
*x* ^2^	–	–	–	–	4.617
*p*	–	–	–	–	0.031

## 4 Discussion

### 4.1 Mixed teaching based on the ADDIE model can improve nursing personnel training effectiveness

The nursing field imposes strict requirements on professional knowledge and practical skills. This is particularly true for new nurses, as their competence and service ability directly affect nursing quality and patient safety (10). Therefore, the training of new nurses is crucial, and it is necessary to establish a nursing training system centered on nurses’ needs and core competencies to enhance their professional quality and service ability (11). The ADDIE model is a practical instructional design model that allows educators to design courses and arrange teaching methods based on learners’ characteristics and needs. It helps identify and address obstacles in educational development, continually improving the quality and effectiveness of teaching (12). The results of this study show that after training, the theoretical knowledge and practical skills of the observation group were significantly higher than those of the control group (P<0.05), consistent with previous studies. Specifically, Sariem et al. (13) successfully developed a leadership and clinical-pharmacy advancement training curriculum for intern pharmacists in Nigeria using the ADDIE model. Despite limited prior exposure, participants showed high levels of preparedness and belief in their capacity to lead in advancing clinical pharmacy practice. Kardosod et al. (14) designed and implemented a blended learning curriculum for surgical nursing practicum using the ADDIE model and a digital notebook application. Both experts and nursing students rated the curriculum highly, indicating its effectiveness in enhancing learning experiences and satisfaction levels. These findings suggest that utilizing digital tools in nursing education, especially during the COVID-19 pandemic, can be beneficial for students’ learning outcomes and overall satisfaction. Furthermore, the study highlights the potential for incorporating similar approaches into nursing curricula globally, contributing to the advancement of online learning platforms in nursing education. In the analysis phase, we systematically analyzed the current status and urgent needs of clinical nursing education from three aspects: learners, learning environment, and learning content. We designed corresponding training courses and teaching methods, ensuring that each stage of pre-class, in-class, and post-class activities was interconnected, thus establishing a complete, systematic, and scientific training system. Such arrangements make nursing education more targeted and effective, enabling new nurses to have clear plans and objectives during training, which helps them integrate the knowledge learned into clinical practice. Teachers also provide evaluation feedback and knowledge reinforcement using various forms such as case analysis, mind maps, and reflective journals, overcoming the previous single face-to-face teaching method. This approach aids in consolidating and deepening new nurses’ knowledge, thereby improving training effectiveness.

### 4.2 Mixed teaching based on the ADDIE model can enhance nursing personnel’s self-learning ability

For new nurses, rapidly mastering clinical nursing knowledge and skills is a key element in adapting to job requirements and establishing a solid foundation for work. Newly trained nurses undergo a transition from student nurses to nursing professionals, lacking theoretical frameworks and work habits, and their educational backgrounds and personal abilities vary widely. Therefore, selecting appropriate training methods is crucial to help them quickly adapt to their job positions (15). The research results show that after training, the observation group’s scores in various dimensions of self-learning ability (learning motivation, planning and implementation, self-management, interpersonal communication) and total scores were significantly higher than those of the control group (*p* < 0.05). The research findings indicate that the observation group, which received training utilizing blended learning based on the ADDIE model, demonstrated significantly higher scores in self-learning ability compared to the control group. Specifically, the observation group exhibited enhanced levels of learning motivation, better planning and implementation skills, improved self-management abilities, and more effective interpersonal communication capabilities. These results suggest that the blended learning approach, combined with the systematic instructional design of the ADDIE model, effectively promotes various aspects of self-directed learning among participants. This is similar to prior research (16) results, which showed that blended-teaching method could better suit some students, depending on their degree of motivation and level of self-directed learning readiness. The reasons for this may be as follows: (1) Mixed learning overcomes the problems of narrow coverage and difficulty in concentrated learning in nursing training. Through online platforms for resource management, new nurses can plan and manage their learning behavior, master progress autonomously, and are not limited by time and space. This allows them to easily access the required information, and difficult points and doubts can be reviewed repeatedly, transforming from passive learning to active learning, promoting personalized self-learning. (2) The training model based on the ADDIE model also plays an important role. Before class, nurses can use online resources to self-study according to learning objectives, identifying weak points in advance. In class, teachers select moderately difficult cases for teaching, simulate clinical scenarios, and empower nurses as active learners. This integrated teaching model can stimulate nurses’ interest in learning, thereby enhancing their self-learning ability (17).

### 4.3 Mixed teaching based on the ADDIE model facilitates the development of nursing personnel’s positive critical thinking skills

The cultivation of critical thinking skills among nursing interns is crucial, as it involves problem-solving techniques and attitudes, influenced by various factors such as educational background and work experience (18). Some studies (19, 20) suggest that critical thinking primarily involves judgment of the authenticity, scientificity, and accuracy of acquired knowledge, requiring rational thinking activities based on personal judgment. In the nursing field, critical thinking refers to the judgment, reflection, reasoning, and decision-making processes regarding complex clinical issues. However, in most medical colleges and universities in China, education for nursing majors still predominantly relies on traditional classroom teaching methods, with basic and specialized courses typically taught separately in chapters and sections. Most clinical nursing instructors are also clinical nursing practitioners facing heavy workloads and pressure, often lacking the energy to contemplate teaching methods and approaches. Consequently, they habitually adhere to traditional teaching methods, focusing on imparting knowledge points. This teacher-centric teaching model accustomed students to passive knowledge acquisition, lacking awareness of independent exploration and innovation. Such teaching models usually result in students mastering only basic theories and fundamental operational skills, while comprehensive qualities and innovative abilities are difficult to effectively cultivate, significantly affecting students’ overall development (21, 22). The research results of this study showed that after training, the observation group’s scores in various dimensions of critical thinking skills (seeking truth, analytical ability, systematic thinking, confidence in critical thinking, thirst for knowledge) and total scores were significantly higher than those of the control group (*p* < 0.05). The findings of this study suggest that the observation group, which underwent training using blended learning based on the ADDIE model, exhibited significantly higher scores in critical thinking skills compared to the control group. Specifically, individuals in the observation group demonstrated greater proficiency in seeking truth, analytical ability, systematic thinking, confidence in critical thinking, and thirst for knowledge. This indicates that the structured approach of blended learning, coupled with the systematic design principles of the ADDIE model, effectively enhances various dimensions of critical thinking among participants. Such improvements signify a deeper engagement with complex issues, a greater ability to analyze information critically, and a more systematic approach to problem-solving, which are essential skills for success in various professional contexts. Our findings are consistent with prior research (23) findings, which showed that blended learning could be effective educational approaches to improve the critical thinking ability of undergraduate nursing students. The reasons for this may be: (1) The mixed teaching model based on the ADDIE model differs from traditional teaching methods in philosophy and implementation. In this teaching model, students are at the center, with problems as the starting point and guidance, inspiring students to autonomously discover issues through clinical cases, followed by independent learning and group discussions under the teacher’s guidance, ultimately solving problems. This learning model itself is the process of building critical thinking, effectively enhancing students’ critical thinking skills. (2) The mixed teaching model based on the ADDIE model focuses on cultivating students’ abilities rather than simply imparting knowledge. It helps students establish a holistic view, emphasizing the integrated application of multidisciplinary knowledge, enhancing students’ perceptual understanding and comprehension of relevant knowledge through research and discussions on issues. (3) In the mixed teaching model based on the ADDIE model, students take the lead and need to maintain a high level of concentration, actively participating in the learning process, which is conducive to cultivating students’ self-learning ability and good thinking habits. At the same time, it provides students with opportunities for teamwork, mutual respect, and communication skills, facilitating the cultivation of positive critical thinking skills.

### 4.4 Mixed teaching based on the ADDIE model enhances nursing personnel’s satisfaction with teaching

In traditional training processes, nurses typically assume the role of listeners, needing to sift and integrate effective information themselves, while instructional teachers follow training plans and processes with minimal interaction. This situation may, to some extent, affect the effectiveness of training and the satisfaction of trainees. This study showed that the teaching satisfaction of the control group was 81.40%, while that of the observation group was 97.73%, with the observation group’s satisfaction significantly higher than that of the control group (*p* < 0.05). The results indicate a significant disparity in teaching satisfaction between the control and observation groups, with the observation group reporting notably higher levels of satisfaction. This suggests that the implementation of blended learning based on the ADDIE model led to a more positive and satisfactory learning experience for participants compared to conventional teaching methods. The statistically significant difference underscores the effectiveness of the innovative teaching approach in enhancing overall satisfaction among learners. This finding highlights the potential of blended learning strategies, particularly those grounded in systematic instructional design models like ADDIE, to positively impact students’ perceptions of teaching quality and effectiveness. This finding is consistent with previous research (24) results, which suggested that blended learning can effectively improve the knowledge and satisfaction of nursing students. The reasons for this may be: (1) Traditional teaching models tend to be teacher-centric, while teaching based on the ADDIE model focuses more on learners’ needs and objectives, making teaching more personalized and standardized. (2) In teaching based on the ADDIE model, online teaching resources are integrated throughout the course, including rich learning materials such as instructional videos, clinical cases, and post-lesson exercises, making the format more engaging and effectively enhancing trainees’ participation and learning outcomes. (3) Teaching innovations based on the concept of smart education have become an inevitable trend. The “Internet+” teaching model breaks the constraints of traditional teaching in terms of time, location, and channels, making learning more flexible and active, thereby increasing trainees’ enthusiasm for learning and further enhancing their satisfaction with teaching.

It is worth noting that despite the beneficial findings regarding the application of mixed teaching based on the ADDIE model in nursing personnel training, there are still some potential shortcomings in this study, such as: (1) Small sample size: This study included only 87 cases, all from the same medical institution, which may limit the universality and reliability of the results. Larger-scale studies may better reflect the effectiveness of mixed teaching based on the ADDIE model in nursing personnel training. (2) Lack of long-term tracking: This study only retrospectively analyzed the results at the end of the training, without conducting long-term follow-up observations. Long-term tracking can better assess the persistence and stability of training effects. (3) Insufficient consideration of other factors: This study did not consider other factors that may affect training effects, such as individual learning abilities and educational backgrounds. Better control of these factors may more accurately reveal the actual impact of mixed teaching on training effects. (4) Potential evaluation bias: The evaluation of teaching satisfaction may suffer from subjective bias because it is based on individuals’ subjective perceptions. More objective evaluation indicators may help accurately assess teaching effectiveness. In summary, although this study provides some valuable results, there are some shortcomings in terms of sample size, tracking time, control of other factors, and objective evaluation.

Future research should focus on several key areas to further enhance our understanding and implementation of mixed teaching based on the ADDIE model in nursing personnel training. Firstly, longitudinal studies are needed to track the long-term effects of this training approach, providing insights into its sustainability and persistence over time. Additionally, comparative studies across diverse healthcare settings and regions would help identify factors influencing its effectiveness and generalizability. Employing mixed methods research, combining quantitative analysis with qualitative insights, can deepen our understanding of nursing personnel’s experiences with mixed teaching, uncovering barriers and areas for improvement. Investigating individualized learning approaches tailored to the unique needs of nursing personnel and exploring the integration of emerging technologies into training programs are also promising avenues for future research. Furthermore, examining the benefits of interprofessional collaboration in nursing training and implementing continuous quality improvement initiatives to refine training design and delivery are essential for ensuring ongoing enhancement of training effectiveness. By addressing these future research directions, we can advance knowledge in nursing personnel training and contribute to the continuous improvement of educational practices in healthcare settings.

## 5 Conclusion

The application of mixed teaching based on the ADDIE model in nursing personnel training yields ideal results. Compared to conventional nursing education and training, mixed teaching based on the ADDIE model can effectively enhance nursing personnel’s self-learning ability, critical thinking skills, mastery of theoretical knowledge, and clinical practice capabilities, thereby increasing their satisfaction with teaching and training.

## Data availability statement

The original contributions presented in the study are included in the article/supplementary material, further inquiries can be directed to the corresponding author.

## Ethics statement

The studies involving humans were approved by Xi’an Qinhuang Hospital, Xi’an, China. The studies were conducted in accordance with the local legislation and institutional requirements. The participants provided their written informed consent to participate in this study.

## Author contributions

RL: Conceptualization, Data curation, Writing – original draft, Writing – review & editing. JL: Funding acquisition, Writing – original draft, Writing – review & editing. XZ: Formal analysis, Investigation, Writing – original draft, Writing – review & editing. DT: Data curation, Project administration, Writing – original draft, Writing – review & editing. YZ: Conceptualization, Writing – original draft, Writing – review & editing.
